# Genetic variants in Forkhead box O1 associated with predisposition to sepsis in a Chinese Han population

**DOI:** 10.1186/s12879-019-4330-7

**Published:** 2019-09-06

**Authors:** Huijuan Wang, Zhaohui Tong, Jia Li, Kun Xiao, Feifei Ren, Lixin Xie

**Affiliations:** 1 Department of Respiratory Medicine, Chinese PLA General Hospital, 28th Fuxing Road, Beijing, 100853 China; 2 Department of Respiratory and Critical Care Medicine, Beijing Chao-Yang Hospital, Beijing Institute of Respiratory Medicine, Capital Medical University, Beijing, 100020 China; 3 Department of Nanlou Respiratory Medicine, Chinese PLA General Hospital, 28th Fuxing Road, Beijing, 100853 China

**Keywords:** Sepsis, Genetic variant, Exome, Forkhead box O1, Single nucleotide polymorphisms

## Abstract

**Background:**

Genetic variant is one of the causes of sepsis patients’ mortality. Now, many studies have identified several SNPs related to sepsis. However, none of these studies were identified in a genome-wide way. We aimed to detect genetic polymorphisms of sepsis patients.

**Methods:**

The blood samples of eight normal controls and ten sepsis patients were collected for whole exome sequencing. Then, Single Nucleotide Polymorphisms (SNPs) were selected according to quality score and number of sepsis patients who had this variants. Synonymous mutations were removed. Genes including these remaining variants were used for functional analyses. After analyses, the remaining SNPs and indels were validated in 149 normal controls and 156 sepsis patients. Finally, serum levels of proteins coded by genes including these SNPs were evaluated.

**Results:**

After whole exome sequencing, 97 SNPs and one indel site were left. Then, functional screening was performed. Only seven SNPs were used for further validation. As a result, the rs2721068 in dominant model and rs17446614 in recessive model were associated with sepsis, and the ORs of these two SNPs were 3.24 (95%CI, 1.25, 8.44) and 0.47 (0.026, 0.88), respectively. These two SNPs were both located in Forkhead box O1 (FOXO1) gene. For rs2721068 (T/T, T/C-C/C) and rs17446614 (A/A-A/G, G/G), serum levels of foxo1 in sepsis patients were both significantly lower in normal controls.

**Conclusions:**

We firstly reported that the rs2721068 and rs17446614 were correlated to genetic predisposition to sepsis.

**Electronic supplementary material:**

The online version of this article (10.1186/s12879-019-4330-7) contains supplementary material, which is available to authorized users.

## Background

Sepsis is a complex disease that can result from any type of infection, and it involves the interplay between pro- and anti-inflammatory mediators and the unpredictable host defense response [1]. Compared to the number of studies that focused on the process of inflammatory response [2, 3], there are fewer studies that focused on genetic predisposition, but there is a representative number. In fact, there have been multiple clinical case reports and many previous clinical retrospective studies that have found that sepsis patients with similar pathogenic infections or with similarly severe conditions often had different outcomes [4]. One of our previous studies showed that patients with sepsis matched by their Sequential Organ Failure Assessment (SOFA) score and Acute Physiology and Chronic Health Evaluation (APACHE II) score had different prognoses [5]. Detection of single nucleotide polymorphisms (SNP) has become an effective method to determine the genetic predisposition to many diseases, including sepsis.

Currently, there are two methods that can be used to identify sepsis-related SNPs. First, many proteins have been shown to be involved in sepsis, and many SNPs are located in the genes that encode these proteins. The interleukin-10 genetic polymorphism rs2227307 and the CXCR2 polymorphism rs1126579 modulate the predisposition to septic shock [6]. Triggering the receptor expressed on myeloid-1 (TREM-1) results in amplifying inflammation and serves as a critical mediator of the inflammatory response in the context of sepsis [7]. The TREM-1 genetic polymorphism rs2234246 was shown to be significantly correlated with the susceptibility to septic shock [8]. In addition to SNPs, insertion/deletion (indel) polymorphisms have also been correlated with sepsis. Serum angiotensin-converting enzyme (CD143) levels are genetically regulated by an indel polymorphism in intron 16 of the CD143 gene [9]. A meta-analysis suggested that indel polymorphisms in the CD143 gene might influence the risk of sepsis, especially pediatric sepsis [10]. In addition to these SNPs and indel sites, many other SNPs have been shown to be related to sepsis, including SNPs located in interleukin 1 receptor antagonist (IL1RN) [11], solute carrier family two member ten (SLC2A10), potassium two pore domain channel subfamily K member 9 (KCNK9) [12], and interleukin-6 (IL-6) [13].

Another method used to identify SNPs related to diseases is whole-exome sequencing. Some rare variants or even novel variants have been identified in patients with genetic diseases or complex diseases with a genetic predisposition. For example, Gao L et al. identified a rare variant (rs55687265) in ATPase phospholipid transporting 8B4 (ATP8B4) as a risk factor for systemic sclerosis using whole-exome sequencing [14]. In addition, some novel candidate genes related to the susceptibility to chronic obstructive pulmonary disease (COPD) were identified using whole-exome sequencing [15].

Hence, in this study, we used whole-exome sequencing for the initial screening of sepsis-related SNPs. After validation, SNPs located in the Forkhead box O1 (FOXO1) gene were proven to be related to sepsis, and the serum levels of FOXO1 in individuals with different FOXO1 genotypes were also evaluated.

## Methods

### Study design

**Fig. 1:**
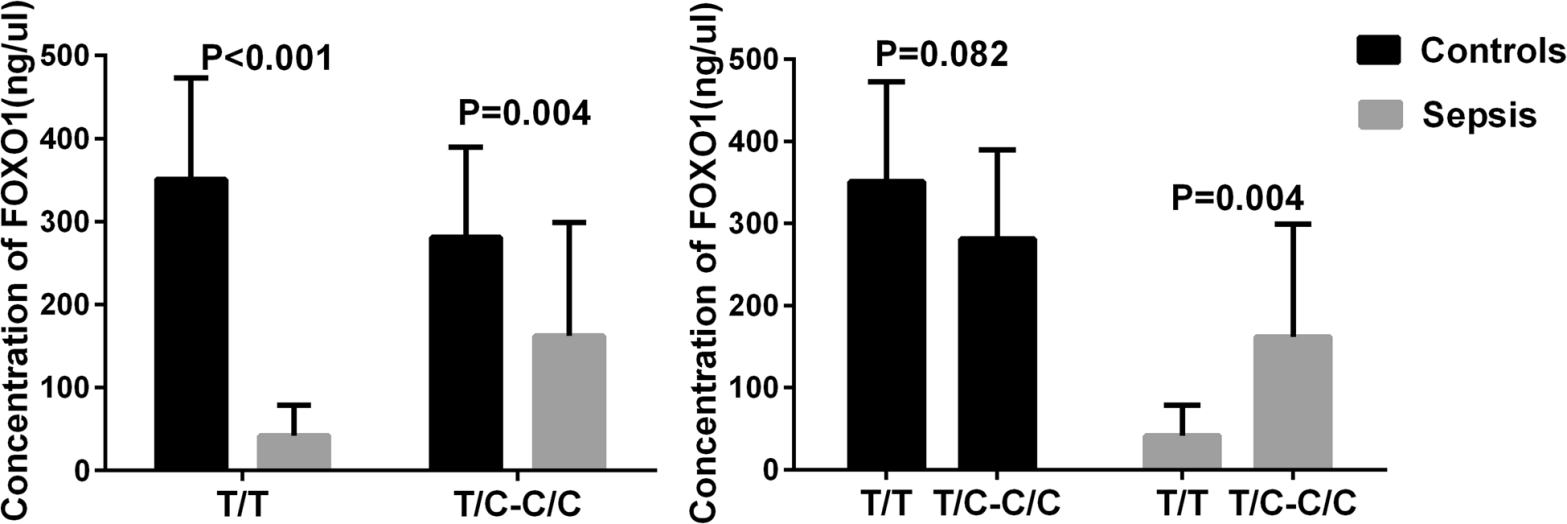
Flow chart of study design

In this study, there were three steps for the SNP identification process. First, ten sepsis patients and eight normal controls were selected for the initial screening of SNPs or indel sites using whole-exome sequencing, and sepsis-related SNPs and indel sites were selected using bioinformatics analysis. Second, the selected SNPs and indel sites were validated in another 156 sepsis patients and 149 normal controls. Third, the protein levels of the genes containing the SNPs evaluated. A flow chart of this process is shown in Fig. 1.

### Ethics

All sepsis patients and normal controls provided written informed consent. This study was also approved by the Committee on Ethics of the Chinese PLA General Hospital (ID: 20111013–009).

### Study subjects and blood sample collection

Whole blood samples were draw from sepsis patients who were admitted to the ICU of the Chinese PLA General Hospital within 24 h. All sepsis patients met the third international consensus definitions for sepsis and septic shock [16]. Sepsis patients who were older than 18 years old were included in this study. Sepsis patients who were immunosuppressed or those who did not receive adequate treatment were excluded. The normal controls were all recruited from the Health Screening Center of the Chinese PLA General Hospital. All blood samples were collected in EDTA tubes, and DNA was isolated for further sequencing.

### Whole-exome sequencing

In order to identify disease-associated genes, whole-exome sequencing was performed using eight normal controls and ten sepsis patients. Human DNA was extracted from peripheral whole blood samples using the TIANamp Blood DNA Kit (Tiangen Biotech Company, Beijing, China). First, extracted DNA was fragmented using the Covaris acoustic system. Then, both ends of these fragments were ligated to adapters. These DNA products were then amplified by ligation-mediated polymerase chain reaction (LM-PCR). After purification, the products were hybridized to the Nimblegen SeqCap EZ Library v3.0 (Roche/NimbleGen, Madison, WI) for enrichment [17]. In order to evaluate the magnitude of enrichment, quantitative PCR (qPCR) was used. These captured libraries were then loaded into a human all-in-one sequencing platform (BGI, Shenzhen, China). High-throughput sequencing was performed to ensure that the average sequencing depth (90×) was met for each sample. Raw reads were processed by Illumina base-calling software 1.7 to test the quality of raw reads and remove polluted reads.

### Bioinformatics analysis

The clean reads were aligned to the NCBI human reference genome using SOAPaligner (soap2.21) [18]. Then, SOAPsnp software (version 1.03) was used to assemble the consensus sequences and call genotypes in the target regions [19]. As for indel sites, BWA was used to map the clean reads onto the reference [20], and we then passed the alignment results to the Genome Analysis Toolkit (GATK) to identify the breakpoints [21]. The low-quality variations were filtered out using the following criteria: (i) variant quality is equal to or larger than 20; (ii) the sequencing depth is between 4 and 1,000,000; (iii) the estimated copy number is no more than two; and (iv) the distance between two SNPs is larger than five. Only mapped reads were used for the subsequent analysis.

### SNP selection

The quality score of the SNPs and indel sites had to be higher than 95% to be included. After removing the synonymous mutations, only the remaining SNPs and indel sites that existed in more than five sepsis patients were selected. Then, the genes of the remaining SNPs and indel sites were analyzed using GO analysis [22] and KEGG analysis [23]. These genes were entered into the GO and KEGG website to obtain enriched GO terms and significant KEGG pathways. The genes that were present in both the enriched GO terms and significant KEGG pathways were used for further analysis. After analysis, the remaining SNPs and indel sites were used for a validation study in a larger sample size.

### Validation of the SNPs

The primers for the selected SNPs were designed using Assay Design 3.1 from the Sequenom Company. In addition, the quality of the primers was checked by matrix-assisted laser desorption/ionization time of flight mass spectrometry (MALDI-TOF-MS). The genotyping reaction was amplified using ABI GeneAmp® 9700 384 Dual (95 °C for 2 min, followed by 45 cycles of 95 °C for 30 s, 56 °C for 30 s, and 72 °C for 60 s and then 72 °C for 5 min). Then, after purification using resin, the products were placed on a chip using a MassARRAY RS1000 nanodispenser and detected using the MassARRAY Compact System. Finally, Typer 4.0 software was used to analyze the data. The genotyping success rate was between 99.2 and 99.6%.

### Enzyme-linked immunosorbent assay

Serum samples from the sepsis patients were also collected. The levels of the proteins encoded by the genes in which the validated SNPs were located were evaluated using an enzyme-linked immunosorbent assay (Elisa) (Antibody-Online) following the manufacturer’s instructions. All assays were performed in duplicate.

### Statistical analysis

The results for non-continuous variables are provided as medians. The Mann-Whitney U-test was used to compare means between two groups. The genotype distributions of all groups were assessed using Hardy-Weinberg equilibrium testing. The frequencies of alleles and genotypes were compared using chi-squared tests or Fisher’s exact two-tailed tests where appropriate, and *p* values were adjusted for the false discovery rate using the Benjamini-Hochberg method. Different models of inheritance were evaluated using SNPStats software (http://bioinfo.iconcologia.net/index.php?module=Snpstats) [24]. Serum levels of FOXO1 were compared using parametric test. Values of *p* < 0.05 were considered statistically significant. All statistical analyses were performed using SPSS software version 22.0 (SPSS, Chicago, USA).

## Results

### Characteristic of the participants included in whole-exome sequencing

Eighteen participants (eight normal controls and ten sepsis patients) were used for the initial whole-exome sequencing analysis, and their clinical data are shown in Table 1. Age (*p* = 0.790) and sex (*p* = 0.681) were matched between the eight normal controls and ten sepsis patients. The ten sepsis patients included five survivors and five non-survivors according to their 28-day mortality rates. The survivors and non-survivors were also matched by age (*p* = 0.917), sex (*p* = 0.738) and severity including APACHE II, SOFA, CRP, PCT.

**Table 1:** Characteristics of the 8 normal controls and 10 sepsis patients used for whole-exome sequencing

Variables	Normal controls (*n* = 8)	Sepsis patients (*n* = 10)			*P* value
		Survivors (*n* = 5)	Non-survivors (*n* = 5)	*P*′ value	
Age (years)	66 (35, 78)	67 (34, 77)	73 (49, 74)	0.917	0.790
Sex (Female/Male)	4/4	2/3	3/2	0.738	0.681
Cause of sepsis (n)					
Lung infection	–	2	2	–	–
Post-operative	–	1	0	–	–
Multiple trauma	–	1	2	–	–
Others	–	1	1	–	–
SOFA score	–	6 (0,7)	8 (5,18)	0.151	–
APACHE II	–	10 (5, 14)	13 (5,23)	0.548	–
CRP (mg/dl)	–	7.2 (1.3, 11.73)	9.23 (2.13, 16.78)	0.548	–
PCT (ng/ml)	–	5.92 (0.05, 9.34)	4.68 (0.1, 64.43)	0.690	–
WBC(×10^9^/l)	–	8.25 (3.01, 35.82)	12.68 (8.8, 17.14)	0.690	–

*SOFA* Sequential Organ Failure Assessment, *APACHE II* Acute physiology and chronic health evaluation, *CRP* C-reactive protein, *PCT* Procalcitonin, *WBC* White blood cells

### Whole-exome sequencing

For these 18 participants, the whole-exome sequencing results showed that a mean of 41,483,912 reads mapped to the target region, and the mean sequencing depth of the region was 69.12×. The average numbers of SNPs and indel sites were 109,379 and 6412, respectively. No indel sites were detected for two sepsis patients (Table 2).

**Table 2:** Summary of whole exome sequencing data of 8 normal controls and 10 sepsis patients

Samples	Reads mapped to target region	Mean depth of region(X)	Coverage of target region(%)	Average read length	Total number of SNPs	Total number of Indels
NC1	50,050,319	79.25	99.26	89.90	109,724	7309
NC2	49,779,034	78.70	99.33	89.88	110,212	7239
NC3	50,051,974	79	99.34	89.90	111,111	7432
NC4	25,687,697	40.45	99.36	89.89	105,453	6979
NC5	4,691,348	74.07	99.31	89.89	111,831	7353
NC6	45,890,953	72.54	99.19	89.90	110,199	7095
NC7	45,813,518	72.39	99.08	89.90	108,477	6929
NC8	31,627,859	49.99	99.44	89.88	107,055	7074
S1	46,780,141	73.56	99.16	89.91	111,024	7190
S2	47,293,254	74.47	99.30	89.91	112,034	0
S3	43,554,210	68.62	99.19	89.91	111,222	7235
S4	51,331,489	81.15	99.24	89.91	107,137	7394
S5	49,780,020	78.21	99.34	89.90	113,155	7264
S6	44,727,205	70.53	99.39	89.91	110,979	7370
S7	30,264,109	47.78	99.46	89.90	104,371	6983
S8	46,511,058	73.56	99.27	89.91	110,268	7231
S9	52,349,603	82.90	99.32	89.91	107,094	7350
S10	30,526,634	48.10	99.39	89.91	107,478	0
Mean	41,483,912	69.12	99.30	89.90	109,379	6412

*NC* Normal control, *S* Sepsis

### Screening of sepsis-related SNPs and indel sites

After sequencing, a total of 34,119 SNPs and indel sites were present in the sepsis patients, and some of these were novel. Several SNPs were present in eight of the ten sepsis patients. After the synonymous mutations were removed, only SNPs that existed in more than five sepsis patients and had a quality score above 95% were selected. Then, there were 97 SNPs and one indel site left, and their detailed information is shown in Additional file 1: Table S1. The genes in which these SNPs and the indel site were located were all entered into Go website and KEGG website. After Go analyses, results showed that there was adenyl nucleotide binding (Go:0030554), adenyl ribonucleotide binding (GO:0032559) and other 22 functional go terms were enrichment with corrected *p* value above 0.05 (Additional file 2: Table S2a). After KEGG analyses, focal adhesion (ko04510), Foxo signaling pathway (ko07201) and other 14 KEGG pathway were enriched (Additional file 2: Table S2b). Then, the common genes that contained enriched GO terms and enriched KEGG pathways were selected. Finally, there were five genes left, including CD1a molecule (CD1A), secreted phosphoprotein 1 (SPP1), collagen type1, alpha2 (COL1A2), serpin peptidase inhibitor, clade A, member 13 (SERPINA13), and FOXO1, and 7 SNPs (rs2269715, rs1126772, rs41317734, rs62464631, rs56952063, rs2721068, rs17446614) were located in these genes. The basic information for these genes and SNPs is shown in Additional file 3: Table S3.

### Validation in a larger sample size

These seven selected SNPs were further validated in 149 normal controls and 156 sepsis patients matched by sex (*p* = 0.567) and age (*p* = 0.543). The clinical data of these participants are shown in Table 3. After validation, the data for these SNPs were subjected to Hardy-Weinberg equilibrium testing. The results showed that the *p* values for the seven SNPs in the normal controls were all higher than 0.05 (Table 4). Then, these seven SNPs were used for further analysis. Comparisons of genotype frequency in different models of inheritance were performed. The results showed that the genotype frequencies of rs2269715 (adjusted *p* value (^a^p) was 0.03) in the recessive model; rs2721068 in the codominant model (^a^*p* = 0.03) and dominant model (^a^*p* = 0.02); and rs17446614 in the codominant model (^a^*p* = 0.046), recessive model (^a^*p* = 0.023), and overdominant model (^a^*p* = 0.013) were significantly different between the sepsis patients and normal controls. The genotype frequency of rs2269715 (^a^*p* = 0.021) was significantly different between survivors and non-survivors (Table 5). Then, the associations of sepsis with rs2269715, rs2721068, and rs17446614 in the different models were further evaluated using SNPstats software. The data showed that only the *p* values of rs2721068 in the codominant and dominant models and the *p* values of rs17446614 in the recessive and overdominant models were below 0.05. However, The OR (95%CI) of rs2721068 in the codominant model was 1.33 (0.78, 2.18) for C/C. Thus, only the dominant model was used for further analysis. Then, the model of inheritance with the lowest values for Akaike’s information criterion (AIC) and Bayesian information criterion (BIC) was selected for rs17446614. Therefore, the dominant model for rs2721068 and recessive model for rs17446614 were selected, and the ORs of these two SNPs were 3.24 (95%CI, 1.25, 8.44) and 0.47 (0.026, 0.88), respectively (Table 6). Then, the association with mortality for rs2269715 was also evaluated, and the *p* values for rs2269715 were all above 0.05 in these four models of inheritance (Additional file 4: Table S4).

**Table 3:** Clinical characteristic of sepsis patients and normal controls used for validation step

Variables	Normal controls (*n* = 149)	Sepsis patients (*n* = 156)	*P* value
Age (years)	66 (35, 78)	60 (29, 82)	0.543
Sex (Female/Male)	70/79	68/88	0.567
Lung infection	–	73 (46.79%)	–
Post-operative	–	33 (21.15%)	–
Multiple trauma	–	27 (17.31%)	–
Others	–	23 (14.75%)	–
SOFA score	–	8 (0, 16)	–
APACHE II	–	20 (8, 34)	–
CRP (mg/dl)	–	9.12 (0.7, 32)	–
PCT (ng/ml)	–	11.32 (0.05, 89.12)	–
WBC(×10^9^/l)	–	11.90 (3.4, 30.67)	–
Mortality(%)	–	45.51%	–

*SOFA* Sequential Organ Failure Assessment, *APACHE II* Acute physiology and chronic health evaluation, *CRP* C-reactive protein, *PCT* Procalcitionin, *WBC* White blood cells

**Table 4:** Functional information and quality control date of the 7 SNPs

SNPs	Gene name	Function	H-W P
rs1126772	CD1A		0.388
rs2269715	SPP1		0.966
rs2721068	COL1A2		0.749
rs17446614	COL1A2		0.904
rs41317734	SERPINA13		0.906
rs62464631	FOXO1		0.821
rs56952063	FOXO1		0.944

*H-W P p* values for Hardy-Weinberg Equilibrium Test of the 7 SNPs in normal controls

**Table 5:** Comparison of different models of inheritance for the 7 selected SNPs

SNP	Model	Genotype	Normal controls	Sepsis patients	*p* value	*p*# value	Survivors (*n* = 85)	Nonsurvivors (*n* = 71)	*p* value	*P*# value
rs1126772	Codominant	A/A	85 (57.05%)	90 (57.69%)	0.999	0.999	47 (55.29%)	44 (61.97%)	0.654	0.658
		A/G	49 (32.88%)	52 (33.33%)			31 (36.47%)	21 (29.58%)		
		G/G	12 (8.05%)	13 (8.33%)			7 (8.24%)	6 (8.45%)		
	Dominant	A/A	85 (57.05%)	90 (57.69%)	0.978	0.978	47 (55.29%)	44 (61.97%)	0.4	0.45
		A/G-G/G	61 (42.95%)	65 (42.31%)			38 (44.71%)	27 (38.03%)		
	Recessive	A/A-A/G	134 (91.95%)	142 (91.67%)	0.958	0.968	7 (8.24%)	6 (8.45%)	0.961	0.963
		G/G	12 (8.05%)	13 (8.33%)			78 (91.76%)	65 (91.55%)		
	Overdominant	A/A-G/G	97 (67.12%)	103 (66.03%)	0.998	0.999	54 (63.53%)	50 (70.42%)	0.363	0.363
		A/G	49 (32.88%)	52 (33.33%)			31 (36.47%)	21 (29.58%)		
rs2269715	Codominant	C/C	59 (39.6%)	58 (37.18%)	0.082	0.092	38 (44.71%)	20 (28.17%)	0.035	0.061
		C/G	70 (46.98%)	79 (50.64%)			35 (41.18%)	44 (61.98%)		
		G/G	19 (12.75%)	8 (5.13%)			12 (14.12%)	7 (9.86%)		
	Dominant	C/C	59 (39.6%)	58 (37.18%)	0.981	0.980	38 (44.71%)	20 (28.17%)	0.033	0.053
		C/G-G/G	89 (60.4%)	87 (55.77%)			47 (55.29%)	51 (71.83%)		
	Recessive	C/C-C/G	129 (87.25%)	137 (87.82%)	0.03	0.03	73 (85.88%)	64 (90.14%)	0.418	0.420
		G/G	19 (12.75%)	8 (5.13%)			12 (14.12%)	7 (9.86%)		
	Overdominant	C/C-G/G	78 (52.35%)	66 (42.31%)	0.219	0.219	50 (58.83%)	27 (38.02%)	0.01	0.021
		C/G	70 (46.98%)	79 (50.64%)			35 (41.18%)	44 (61.98%)		
rs2721068	Codominant	T/T	7 (4.7%)	19 (12.18%)	0.03	0.03	12 (14.12%)	7 (9.86%)	0.665	0.667
		T/C	58 (38.93%)	66 (42.31%)			34 (40.0%)	32 (45.07%)		
		C/C	84 (56.38%)	71 (45.51%)			39 (45.88%)	32 (45.07%)		
	Dominant	T/T	7 (4.7%)	19 (12.18%)	0.019	0.02	12 (14.12%)	7 (9.86%)	0.418	0.428
		T/C-C/C	142 (95.3%)	137 (87.82%)			73 (85.88%)	64 (90.14%)		
	Recessive	T/T-T/C	65 (43.62%)	85 (54.49%)	0.058	0.058	46 (54.12%)	39 (54.93%)	0.919	0.920
		C/C	84 (56.38%)	71 (45.51%)			39 (45.88%)	32 (45.07%)		
	Overdominant	T/T-C/C	92 (61.07%)	90 (57.69%)	0.517	0.520	51 (60.0%)	39 (54.93%)	0.523	0.525
		T/C	58 (38.93%)	66 (42.31%)			34 (40.0%)	32 (45.07%)		
rs17446614	Codominant	A/A	2 (1.34%)	3 (1.92%)	0.045	0.046	1 (1.18%)	2 (2.82%)	0.679	0.680
		A/G	35 (23.49%)	20 (12.82%)			10 (11.76%)	10 (14.08%)		
		G/G	110 (73.83%)	133 (85.25%)			74 (87.06%)	59 (83.10%)		
	Dominant	A/A	2 (1.34%)	3 (1.92%)	0.701	0.701	1 (1.18%)	2 (2.82%)	0.458	0.460
		A/G-G/G	145 (97.32%)	153 (98.08%)			84 (98.82%)	69 (97.18%)		
	Recessive	A/A-A/G	37 (24.84%)	23 (14.75%)	0.023	0.023	11 (12.94%)	12 (16.90%)	0.487	0.489
		G/G	110 (73.83%)	133 (85.25%)			74 (87.06%)	59 (83.10%)		
	Overdominant	A/A-G/G	112 (75.17%)	136 (87.18%)	0.013	0.013	75 (88.24%)	61 (85.92%)	0.666	0.667
		A/G	35 (23.49%)	20 (12.82%)			10 (11.76%)	10 (14.08%)		
rs41317734	Codominant	C/C	121 (81.21%)	133 (85.25%)	0.585	0.589	73 (85.88%)	60 (84.51%)	0.6	0.63
		C/T	26 (17.45%)	22 (14.10%)			11 (12.94%)	11 (15.49%)		
		T/T	2 (1.34%)	1 (0.64%)			1 (1.18%)	0 (0.00%)		
	Dominant	C/C	121 (81.21%)	133 (85.25%)	0.344	0.367	73 (85.88%)	60 (84.51%)	0.809	0.810
		C/T-T/T	28 (18.79%)	23 (14.75%)			12 (14.12%)	11 (15.49%)		
	Recessive	C/C-C/T	147 (98.66%)	155 (99.36%)	0.535	0.535	84 (98.82%)	71 (100%)	0.359	0.360
		T/T	2 (1.34%)	1 (0.64%)			1 (1.18%)	0 (0.00%)		
	Overdominant	C/C-T/T	123 (82.55%)	134 (85.90%)	0.422	0.435	74 (87.06%)	60 (84.51%)	0.648	0.659
		C/T	26 (17.45%)	22 (14.10%)			11 (12.94%)	11 (15.49%)		
rs62464631	Codominant	A/A	2 (1.34%)	1 (0.64%)	0.555	0.556	1 (1.18%)	0 (0.00%)	0.6	0.61
		A/G	26 (17.45%)	22 (14.10%)			11 (12.94%)	11 (15.49%)		
		G/G	119 (79.87%)	133 (85.25%)			73 (85.88%)	60 (84.51%)		
	Dominant	A/A	2 (1.34%)	1 (0.64%)	0.527	0.528	1 (1.18%)	0 (0.00%)	0.359	0.359
		A/G-G/G	145 (97.32%)	155 (99.36%)			84 (98.82%)	71 (100%)		
	Recessive	A/A-A/G	28 (18.79%)	23 (14.75%)	0.317	0.318	12 (14.12%)	11 (15.49%)	0.809	0.812
		G/G	119 (79.87%)	133 (85.25%)			73 (85.88%)	60 (84.51%)		
	Overdominant	A/A-G/G	121 (81.21%)	134 (85.90%)	0.393	0.393	74 (87.06%)	60 (84.51%)	0.648	0.648
		A/G	26 (17.45%)	22 (14.10%)			11 (12.94%)	11 (15.49%)		
rs56952063	Codominant	C/C	10 (6.71%)	7 (4.49%)	0.419	0.420	3 (3.53%)	4 (5.63%)	0.676	0.678
		C/T	54 (36.24%)	67 (42.95%)			35 (41.18%)	32 (45.07%)		
		T/T	84 (56.38%)	82 (43.10%)			47 (55.29%)	35 (49.30%)		
	Dominant	C/C	10 (6.71%)	7 (4.49%)	0.389	0.389	3 (3.53%)	4 (5.63%)	0.527	0.527
		C/T-T/T	138 (92.62%)	149 (95.51%)			82 (96.47%)	67 (94.37%)		
	Recessive	C/C-C/T	64 (42.96%)	74 (56.90%)	0.463	0.470	38 (44.71%)	36 (50.70%)	0.455	0.456
		T/T	84 (56.38%)	82 (43.10%)			47 (55.29%)	35 (49.30%)		
	Overdominant	C/C-T/T	94 (63.09%)	89 (57.05%)	0.25	0.26	50 (58.82%)	39 (54.93%)	0.625	0.626
		C/T	54 (36.24%)	67 (42.95%)			35 (41.18%)	32 (45.07%)		

*P* value: Chi-squared test or Fisher’s exact two-tailed tests; *p*# value: False discovery rate adjusted *p* value using the Benjamini-Hochberg method

**Table 6:** The 7 selected SNPs association with sepsis (*n* = 305, adjusted by sex and age)

SNP	Model	Genotype	Normal controls	Sepsis patients	OR (95% CI)	*P* value	AIC	BIC
rs2269715	Codominant	C/C	59 (39.6%)	58 (37.18%)	1	0.888	− 445.35	− 429.95
		C/G	70 (46.98%)	79 (50.64%)	1.08 (0.57, 2.04)			
		G/G	19 (12.75%)	8 (5.13%)	1.16 (0.62, 2.17)			
	Dominant	C/C	59 (39.6%)	58 (37.18%)	1	0.91	−445.40	− 430.00
		C/G-G/G	89 (60.4%)	87 (55.77%)	0.97 (0.61, 1.56)			
	Recessive	C/C-C/G	129 (87.25%)	137 (87.82%)	1	0.69	−445.42	−430.02
		G/G	19 (12.75%)	8 (5.13%)	1.12 (0.63, 1.99)			
	Overdominant	C/C-G/G	78 (52.35%)	66 (42.31%)	1	0.79	−445.50	−430.10
		C/G	70 (46.98%)	79 (50.64%)	0.94 (0.59, 1.49)			
rs2721068	Codominant	T/T	7 (4.7%)	19 (12.18%)	1	0.028	−451.43	− 436.03
		T/C	58 (38.93%)	66 (42.31%)	3.67 (1.38, 9.77)			
		C/C	84 (56.38%)	71 (45.51%)	1.33 (0.78, 2.18)			
	Dominant	T/T	7 (4.7%)	19 (12.18%)	1	0.016	−451.24	−435.84
		T/C-C/C	142 (95.3%)	137 (87.82%)	3.24 (1.25, 8.44)			
	Recessive	T/T-T/C	65 (43.62%)	85 (54.49%)	1	0.059	− 448.12	−432.72
		C/C	84 (56.38%)	71 (45.51%)	1.57 (0.98, 2.50)			
	Overdominant	T/T-C/C	92 (61.07%)	90 (57.69%)	1	0.719	− 444.45	−429.05
		T/C	58 (38.93%)	66 (42.31%)	0.92 (0.58, 1.45)			
rs17446614	Codominant	A/A	2 (1.34%)	3 (1.92%)	1	0.061	− 452.91	− 437.51
		A/G	35 (23.49%)	20 (12.82%)	0.41 (0.04, 4.58)			
		G/G	110 (73.83%)	133 (85.25%)	0.48 (0.25, 0.90)			
	Dominant	A/A	2 (1.34%)	3 (1.92%)	1	0.545	−446.91	− 431.51
		A/G-G/G	145 (97.32%)	153 (98.08%)	0.47 (0.04, 5.33)			
	Recessive	A/A-A/G	37 (24.84%)	23 (14.75%)	1	0.018	−453.18	−437.78
		G/G	110 (73.83%)	133 (85.25%)	0.47 (0.026, 0.88)			
	Overdominant	A/A-G/G	112 (75.17%)	136 (87.18%)	1	0.028	−452.61	−437.21
		A/G	35 (23.49%)	20 (12.82%)	1.86 (1.07, 3.23)			

*OR* Odd ratio, *CI* Confidential interval, *AIC* Akaike’s Information Criterion, *BIC* Bayesian Information Criterion

### Foxo1 levels

**Fig. 2:**
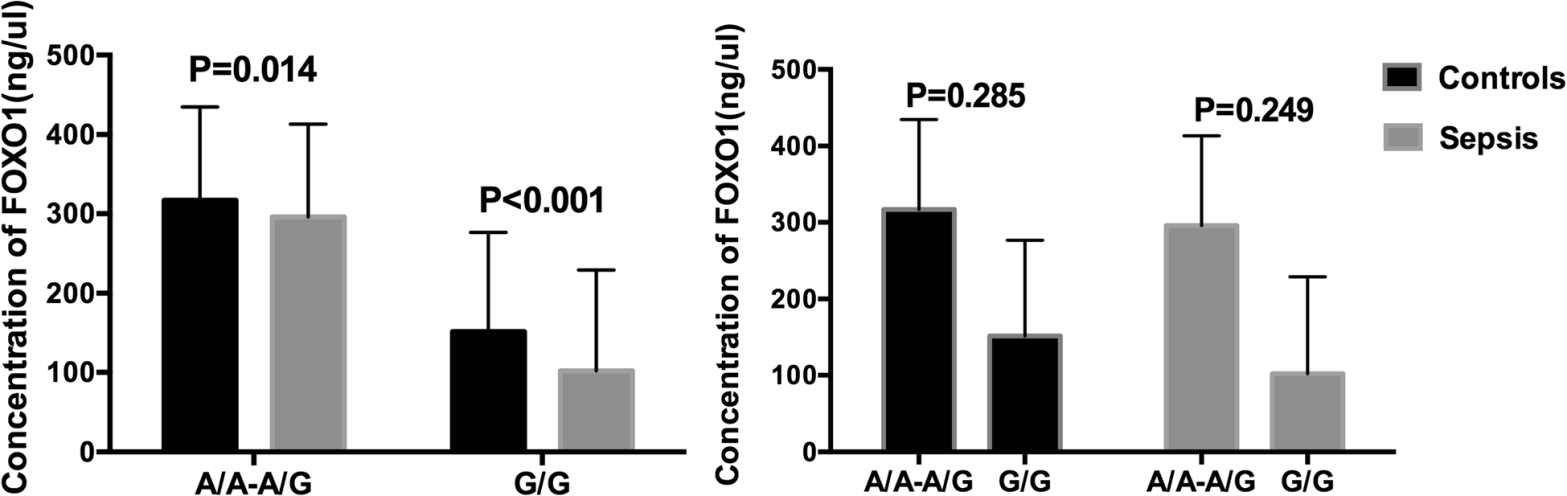
Foxo1 serum levels of rs2721068 in different genotypes. **a** Comparison of foxo1 serum levels between sepsis patients (*n* = 30) and normal controls (*n* = 30) in different genotypes. **b** Serum levels of Foxo1 between different genotypes in sepsis patients (*n* = 30) and normal controls (*n* = 30)

**Fig. 3:**
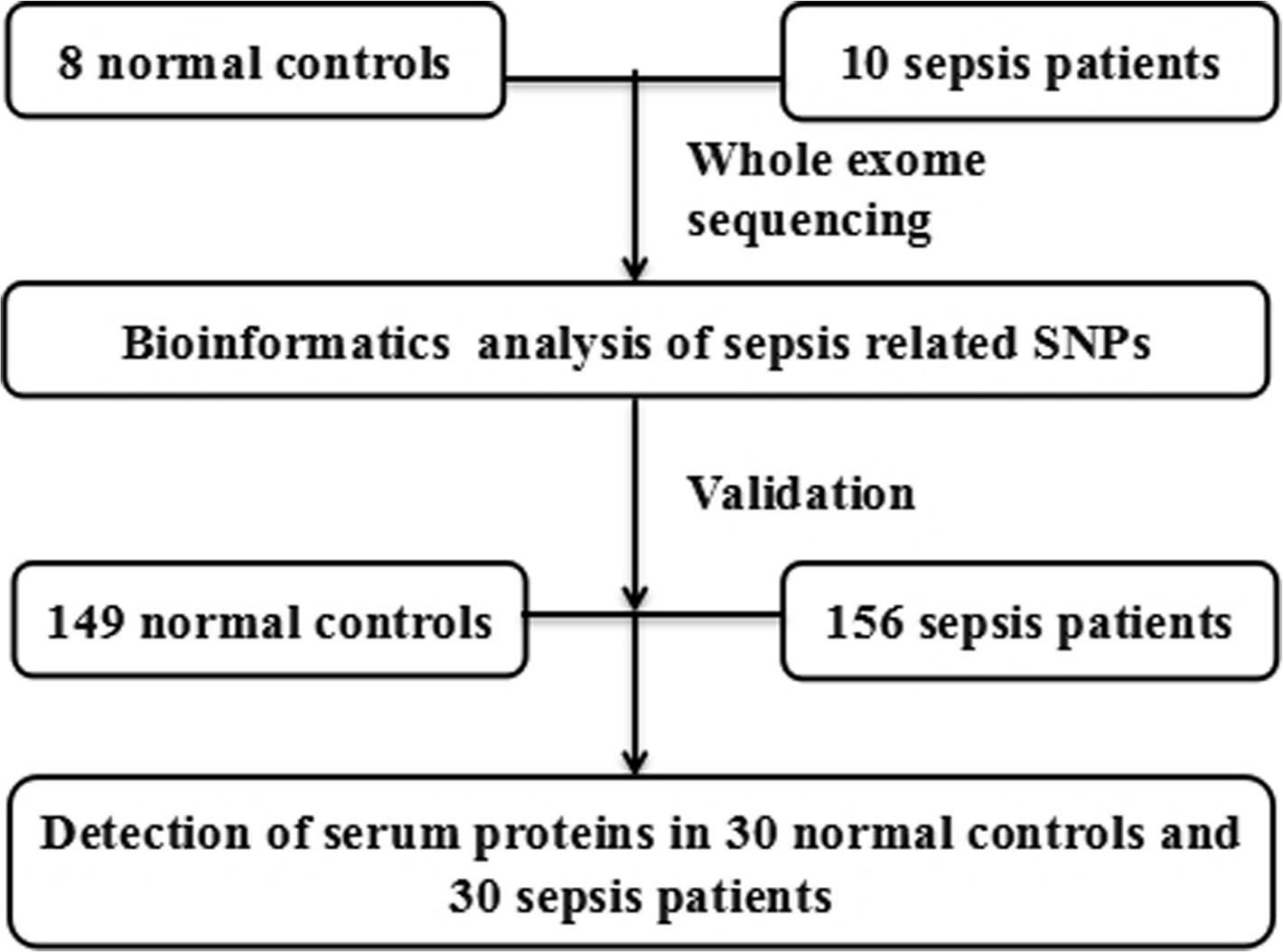
Foxo1 serum levels of rs17446614 in different genotypes. **a** Comparison of foxo1 serum levels between sepsis patients (*n* = 30) and normal controls (*n* = 30) in different genotypes. **b** Serum levels of Foxo1 between different genotypes in sepsis patients (*n* = 30) and normal controls (*n* = 30)

According to our previous sequencing and validation results, rs2721068 in a dominant model and rs17446614 in a recessive model were associated with sepsis. Both of these SNPs are located in the FOXO1 gene. Next, we evaluated the serum levels of FOXO1 in 30 normal controls and 30 sepsis patients. For rs2721068, the levels of FOXO1 in sepsis patients with the T/T and T/C-C/C genotypes were significantly lower than in the normal controls (*p* < 0.001 and *p* = 0.004, respectively). The serum levels of FOXO1 in sepsis patients with the T/C-C/C genotype were significantly higher than in sepsis patients with the T/T genotype (*p* = 0.002) (Fig. 2). For rs17446614, the levels of FOXO1 in sepsis patients with the A/A-A/G and G/G genotypes were significantly lower than in the normal controls (*p* = 0.014 and *p* < 0.001, respectively). In addition, there were no significant differences between the two genotype in the two groups (Fig. 3).

## Discussion

In recent years, although the methods of treatment have been frequently updated and refined, sepsis and septic shock have remained the most common causes of death in intensive care units (ICUs) [25]. Hence, many studies have focused on finding treatment targets for sepsis. In this study, we found that rs2721068 and rs17446614 in the FOXO1 gene were correlated with the genetic predisposition to sepsis after conducting whole-exome sequencing and validation analyses. The serum levels of FOXO1 in sepsis patients with any genotype were significantly lower than in normal controls.

FOXO1 has been shown to be involved in muscle atrophy [26] and insulin resistance [27]. Crossland et al. demonstrated that Akt/FOXO signaling played a role in both protein loss and the impairment of muscle carbohydrate oxidation during sepsis in rodent skeletal muscle [28]. Inhibition of FOXO transcriptional activity was shown to prevent muscle fiber atrophy [29]. One of our previous studies showed that FOXO1 was a target gene of miR-223 and miR-15a/16, which are involved in the pathway of lymphocyte apoptosis during sepsis (data not shown). Another study by our group reported that the serum levels of FOXO1 in normal controls were significantly higher than in sepsis patients, and the values of FOXO1 were also significantly different between survivors and non-survivors according to 28-day mortality [30]. Hence, FOXO1 is involved in the mechanism of sepsis through many different pathways.

The FOXO1a haplotype [C-C-G-A-A-A] has a protective effect on the development of type two diabetes [31]. Two FOXO1a SNPs, rs2755209 and rs2755213, were shown to be associated with female longevity [32]. The rs10507486 and rs2297627 FOXO1 SNPs were shown to be associated with carotid atherosclerosis [33]. The rs17592236 SNP is a target site of the human miRNA miR-137, and the rs17592236 polymorphism is associated with a lower hepatocellular carcinoma hereditary susceptibility likely through modulating the binding affinity of miR-137 to the 3’UTR in FOXO1 messenger RNA [34]. However, to date, no study has shown whether FOXO1 SNPs are correlated with sepsis. Our study demonstrated for the first time that the rs2721068 and rs17446614 FOXO1 SNPs are correlated with the genetic predisposition to sepsis. However, a previous study reported that the rs2721068 and rs17446614 SNPs within the FOXO1 gene affected insulin secretion and glucose tolerance and were associated with an increased risk of type two diabetes [35].

Rs2721068 and rs17446614 are both located in intron regions of FOXO1. We searched against expression Quantitative Trait Loci (eQTL) [36], and no information about the relation between the two SNPs and expression of FOXO1 was obtained. However, there still were some indirect evidences. For rs2721068, this SNP was located in long interspersed repeat elements (LINE) of the genome and research reported that repeat elements can regulate expression of gene by cis/trans regulator [37]. In addition, there were copy number variants (CNVs) in the region where rs2721068 were located in other diseases, including autism [38], cleft palate [39]. For rs17446614, this SNP was located in (short interspersed repeat elements) SINE of the genome and CNVs were also existed in this region in other disease [40].

In our study, there were several novel findings. We first used whole-exome sequencing to screen sepsis-related SNPs. Sepsis is a complex disease, and one or two SNPs alone cannot depict the genetic variation of sepsis. Thus, using a gene-wide approach seems more reasonable. The rs2721068 and rs17446614 SNPs were shown for the first time to correlate to sepsis, and they were also the first SNPs in the FOXO1 gene to be associated with sepsis.

However, there were still several limitations in our study. First, sepsis is a complex disease, and the sample size used for whole-exome sequencing and genotyping was relatively small. Thus, many other SNPs might have been missed, which is the major limitation of our study. Therefore, as a next step, a larger number of sepsis patients will be recruited for whole-exome sequencing in future studies. Second, normal controls used in this study were all healthy controls instead of critical care patients without sepsis, so many parameters like infection were not matched. Third, only a few SNPs were selected from whole exome sequencing for validation and other SNP which were also different between these two groups were not evaluated. There were no significant differences of FOXO1 levels between the two genotype. Finally, our previous study first reported that serum FOXO1 levels were lower in sepsis patients than in normal controls [30], which is consistent with our present results. However, no significant difference in serum FOXO1 levels was found between survivors and non-survivors, indicating that other FOXO1 SNPs need to be evaluated.

As a next step, additional SNPs in FOXO1 will be analyzed, and additional studies are needed to determine the roles of rs2721068 and rs174466614 in the sepsis pathway.

## Conclusion

We reported for the first time that the rs2721068 in dominant model and rs17446614 in recessive model were correlated to sepsis and serum levels of FOXO1 in these genotypes were all significantly higher in normal controls than in sepsis patients.

## Additional files

Additional file 1: **Table S1.** The detail information of sepsis special SNPs after initial screening. After whole exome sequencing, synonymous mutations were removed. SNPs only existed in more than 5 sepsis patients and quality score of these SNPs were higher than 95% were selected. The excel file of Table S1 has been submitted as a separate supplementary dataset. (XLS 119 kb)

Additional file 2: **Table S2.** a. Rich terms for function ontology of genes selected by screened SNPs. b Rich pathways of KEGG analysis of genes selected by screened SNPs. (DOCX 17 kb)

Additional file 3: **Table S3.** The information of SNPs selected for further validation. (DOCX 14 kb)

Additional file 4: **Table S4.** rs2269715 association with mortality of sepsis patients (n=156, adjusted by sex and age). (DOCX 14 kb)

## Data Availability

The datasets analyzed are available from the corresponding author on reasonable request.

## Abbreviations

AIC: Akaike’s information criterion
APACHE: Acute Physiology and Chronic Health Evaluation
ATP8B4: ATPase phospholipid transporting 8B4
BIC: Bayesian information criterion
CD1A: CD1a molecule
CNVs: Copy number variants
COL1A2: Collagen type1, alpha2
COPD: Chronic obstructive pulmonary disease
e eQTL: Expression Quantitative Trait Loci
FOXO1: Forkhead box O1
GATK: Genome Analysis Toolkit
IL1RN: Interleukin 1 receptor antagonist
IL-6: Interleukin-6
KCNK9: Potassium two pore domain channel subfamily K member 9
l LM-PCR: Igation-mediated polymerase chain reaction
LINE: Long interspersed repeat elements
SERPINA13: Serpin peptidase inhibitor, clade A, member 13
SLC2A10: Solute carrier family 2 member 10
SNP: Single nucleotide polymorphisms
SOFA: Sequential Organ Failure Assessment
SPP1: Secreted phosphoprotein 1
TREM-1: Triggering the receptor expressed on myeloid-1

## References

[1] Abu-Maziad A, Schaa K, Bell EF, Dagle JM, Cooper M, Marazita ML, Murray JC (2010). Role of polymorphic variants as genetic modulators of infection in neonatal sepsis. Pediatr Res.

[2] Cao J, Xu F, Lin S, Tao X, Xiang Y, Lai X, Zhang L (2015). IL-35 is elevated in clinical and experimental sepsis and mediates inflammation. Clin Immunol.

[3] Chiswick EL, Mella JR, Bernardo J, Remick DG (2015). Acute-phase deaths from murine Polymicrobial Sepsis are characterized by innate immune suppression rather than exhaustion. J Immunol.

[4] Maslove DM, Wong HR (2014). Gene expression profiling in sepsis: timing, tissue, and translational considerations. Trends Mol Med.

[5] Wang H, Zhang P, Chen W, Feng D, Jia Y, Xie L (2012). Serum microRNA signatures identified by Solexa sequencing predict sepsis patients' mortality: a prospective observational study. PLoS One.

[6] Cardoso CP, de Oliveira AJ, Botoni FA, Rezende IC, Alves-Filho JC, Cunha Fde Q, Estanislau Jde A, Magno LA, Rios-Santos F (2015). Interleukin-10 rs2227307 and CXCR2 rs1126579 polymorphisms modulate the predisposition to septic shock. Mem Inst Oswaldo Cruz.

[7] Klesney-Tait J, Turnbull IR, Colonna M (2006). The TREM receptor family and signal integration. Nat Immunol.

[8] Peng LS, Li J, Zhou GS, Deng LH, Yao HG (2015). Relationships between genetic polymorphisms of triggering receptor expressed on myeloid cells-1 and septic shock in a Chinese Han population. World J Emerg Med.

[9] Villard E, Tiret L, Visvikis S, Rakotovao R, Cambien F, Soubrier F (1996). Identification of new polymorphisms of the angiotensin I-converting enzyme (ACE) gene, and study of their relationship to plasma ACE levels by two-QTL segregation-linkage analysis. Am J Hum Genet.

[10] Yang H, Wang Y, Liu L (2014). Hu Q: increased susceptibility of sepsis associated with CD143 deletion/insertion polymorphism in Caucasians: a meta analysis. Int J Clin Exp Pathol.

[11] Meyer NJ, Ferguson JF, Feng R, Wang F, Patel PN, Li M, Xue C, Qu L, Liu Y, Boyd JH (2014). A functional synonymous coding variant in the IL1RN gene is associated with survival in septic shock. Am J Respir Crit Care Med.

[12] Hoggart CJ, Venturini G, Mangino M, Gomez F, Ascari G, Zhao JH, Teumer A, Winkler TW, Tsernikova N, Luan J (2014). Novel approach identifies SNPs in SLC2A10 and KCNK9 with evidence for parent-of-origin effect on body mass index. PLoS Genet.

[13] Zidan HE, Elbehedy RM, Azab SF (2014). IL6-174 G/C gene polymorphism and its relation to serum IL6 in Egyptian children with community-acquired pneumonia. Cytokine.

[14] Gao L, Emond MJ, Louie T, Cheadle C, Berger AE, Rafaels N, Vergara C, Kim Y, Taub MA, Ruczinski I (2016). Identification of rare variants in ATP8B4 as a risk factor for systemic sclerosis by whole-exome sequencing. Arthritis Rheumatol.

[15] Bruse S, Moreau M, Bromberg Y, Jang JH, Wang N, Ha H, Picchi M, Lin Y, Langley RJ, Qualls C (2016). Whole exome sequencing identifies novel candidate genes that modify chronic obstructive pulmonary disease susceptibility. Human Genomics.

[16] Singer M, Deutschman CS, Seymour CW (2016). The third international consenus definitions for sepsis and septic shock (sepsis-3). JAMA.

[17] Schorderet DF, Iouranova A, Favez T, Tiab L, Escher P (2013). IROme, a new high-throughput molecular tool for the diagnosis of inherited retinal dystrophies. Biomed Res Int.

[18] Li R, Yu C, Li Y, Lam TW, Yiu SM, Kristiansen K, Wang J (2009). SOAP2: an improved ultrafast tool for short read alignment. Bioinformatics.

[19] Li R, Li Y, Fang X, Yang H, Wang J, Kristiansen K, Wang J (2009). SNP detection for massively parallel whole-genome resequencing. Genome Res.

[20] Li H, Durbin R (2010). Fast and accurate long-read alignment with burrows-wheeler transform. Bioinformatics.

[21] McKenna A, Hanna M, Banks E, Sivachenko A, Cibulskis K, Kernytsky A, Garimella K, Altshuler D, Gabriel S, Daly M (2010). The genome analysis toolkit: a MapReduce framework for analyzing next-generation DNA sequencing data. Genome Res.

[22] Ashburner M, Ball CA, Blake JA, Botstein D, Butler H, Cherry JM, Davis AP, Dolinski K, Dwight SS, Eppig JT (2000). Gene ontology: tool for the unification of biology. Gene Ontol Consortium Nat Genetics.

[23] Kanehisa M, Goto S, Kawashima S, Okuno Y, Hattori M (2004). The KEGG resource for deciphering the genome. Nucleic Acids Res.

[24] Sole X, Guino E, Valls J, Iniesta R, Moreno V (2006). SNPStats: a web tool for the analysis of association studies. Bioinformatics.

[25] Ma G, Wang H, Mo G, Cui L, Li Y, Shao Y, Liu X, Xie Y, Li J, Fu J (2014). The Pro12Ala polymorphism of PPAR-gamma gene is associated with Sepsis disease severity and outcome in Chinese Han population. PPAR Res.

[26] Sandri M, Sandri C, Gilbert A, Skurk C, Calabria E, Picard A, Walsh K, Schiaffino S, Lecker SH, Goldberg AL (2004). Foxo transcription factors induce the atrophy-related ubiquitin ligase atrogin-1 and cause skeletal muscle atrophy. Cell.

[27] Kim YI, Lee FN, Choi WS, Lee S, Youn JH (2006). Insulin regulation of skeletal muscle PDK4 mRNA expression is impaired in acute insulin-resistant states. Diabetes.

[28] Crossland H, Constantin-Teodosiu D, Gardiner SM, Constantin D, Greenhaff PL (2008). A potential role for Akt/FOXO signalling in both protein loss and the impairment of muscle carbohydrate oxidation during sepsis in rodent skeletal muscle. J Physiol.

[29] Reed SA, Sandesara PB, Senf SM, Judge AR (2012). Inhibition of FoxO transcriptional activity prevents muscle fiber atrophy during cachexia and induces hypertrophy. FASEB J.

[30] Wang HJ, Wang BZ, Zhang PJ, Deng J, Zhao ZR, Zhang X, Xiao K, Feng D, Jia YH, Liu YN (2014). Identification of four novel serum protein biomarkers in sepsis patients encoded by target genes of sepsis-related miRNAs. Clin Sci.

[31] Bottcher Y, Tonjes A, Enigk B, Scholz GH, Bluher M, Stumvoll M, Kovacs P (2007). A SNP haplotype of the forkhead transcription factor FOXO1A gene may have a protective effect against type 2 diabetes in German Caucasians. Diabetes Metab.

[32] Li Y, Wang WJ, Cao H, Lu J, Wu C, Hu FY, Guo J, Zhao L, Yang F, Zhang YX (2009). Genetic association of FOXO1A and FOXO3A with longevity trait in Han Chinese populations. Hum Mol Genet.

[33] Kedenko L, Lamina C, Kedenko I, Kollerits B, Kiesslich T, Iglseder B, Kronenberg F, Paulweber B (2014). Genetic polymorphisms at SIRT1 and FOXO1 are associated with carotid atherosclerosis in the SAPHIR cohort. BMC Med Gen.

[34] Tan C, Liu S, Tan S, Zeng X, Yu H, Li A, Bei C, Qiu X (2015). Polymorphisms in microRNA target sites of forkhead box O genes are associated with hepatocellular carcinoma. PLoS One.

[35] Mussig K, Staiger H, Machicao F, Stancakova A, Kuusisto J, Laakso M, Thamer C, Machann J, Schick F, Claussen CD (2009). Association of common genetic variation in the FOXO1 gene with beta-cell dysfunction, impaired glucose tolerance, and type 2 diabetes. J Clin Endocrinol Metab.

[36] Michaelson JJ, Loguercio S, Beyer A (2009). Detection and interpretation of expression quantitative trait loci (eQTL). Methods.

[37] Bujko M, Musialik E, Olbromski R, Przestrzelska M, Libura M, Pastwinska A, Juszczynski P, Zwierzchowski L, Baranowski P, Siedlecki JA (2014). Repetitive genomic elements and overall DNA methylation changes in acute myeloid and childhood B-cell lymphoblastic leukemia patients. Int J Hematol.

[38] de Smith AJ, Walters RG, Coin LJ, Steinfeld I, Yakhini Z, Sladek R, Froguel P, Blakemore AI (2008). Small deletion variants have stable breakpoints commonly associated with alu elements. PLoS One.

[39] Toth G, Zraly CB, Thomson TL, Jones C, Lapetino S, Muraskas J, Zhang J, Dingwall AK (2011). Congenital anomalies and rhabdoid tumor associated with 22q11 germline deletion and somatic inactivation of the SMARCB1 tumor suppressor. Genes Chromosomes Cancer.

[40] Satovic E, Plohl M (2013). Tandem repeat-containing MITEs in the clam Donax trunculus. Genome Biol Evol.

