# Supplementary material for: Genetic variants in Forkhead box O1 associated with predisposition to sepsis in a Chinese Han population
Source: BMC Infect Dis. 2019 Sep 6;19:781. doi: 10.1186/s12879-019-4330-7 (PMC6731606; doi:10.1186/s12879-019-4330-7)
Supplement: Supplementary file 2 — Table S2. a. Rich terms for function ontology of genes selected by screened SNPs. b Rich pathways of KEGG analysis of genes selected by screened SNPs. (DOCX 17 kb) [file 12879_2019_4330_MOESM2_ESM.docx]

**Supplementary Table S2a.** **Rich terms for function ontology of genes selected by screened SNPs.**

| **Gene Ontology terms** | **GO Number** | **Corrected P-value** |
| --- | --- | --- |
| [Adenyl nucleotide binding](http://amigo.geneontology.org/cgi-bin/amigo/go.cgi?action=query&view=query&query=GO:0030554&search_constraint=terms) | GO:0030554 | 1.17e-13 |
| [Adenyl ribonucleotide binding](http://amigo.geneontology.org/cgi-bin/amigo/go.cgi?action=query&view=query&query=GO:0032559&search_constraint=terms) | GO:0032559 | 1.42e-12 |
| [Nucleoside binding](http://amigo.geneontology.org/cgi-bin/amigo/go.cgi?action=query&view=query&query=GO:0001882&search_constraint=terms) | GO:0001882 | 1.51e-12 |
| [Purine nucleoside binding](http://amigo.geneontology.org/cgi-bin/amigo/go.cgi?action=query&view=query&query=GO:0001883&search_constraint=terms) | GO:0001883 | 1.78e-12 |
| [Purine nucleotide binding](http://amigo.geneontology.org/cgi-bin/amigo/go.cgi?action=query&view=query&query=GO:0017076&search_constraint=terms) | GO:0017076 | 4.74e-07 |
| [Nucleotide binding](http://amigo.geneontology.org/cgi-bin/amigo/go.cgi?action=query&view=query&query=GO:0000166&search_constraint=terms) | GO:0000166 | 2.05e-06 |
| [Ribonucleotide binding](http://amigo.geneontology.org/cgi-bin/amigo/go.cgi?action=query&view=query&query=GO:0032553&search_constraint=terms) | GO:0032553 | 3.94e-06 |
| [Purine ribonucleotide binding](http://amigo.geneontology.org/cgi-bin/amigo/go.cgi?action=query&view=query&query=GO:0032555&search_constraint=terms) | GO:0032555 | 3.94e-06 |
| [Nucleoside-triphosphatase regulator activity](http://amigo.geneontology.org/cgi-bin/amigo/go.cgi?action=query&view=query&query=GO:0060589&search_constraint=terms) | GO:0060589 | 4.57e-06 |
| [GTPase regulator activity](http://amigo.geneontology.org/cgi-bin/amigo/go.cgi?action=query&view=query&query=GO:0030695&search_constraint=terms) | GO:0030695 | 7.39e-06 |
| [Small GTPase regulator activity](http://amigo.geneontology.org/cgi-bin/amigo/go.cgi?action=query&view=query&query=GO:0005083&search_constraint=terms) | GO:0005083 | 2.24e-05 |
| [Ion transmembrane transporter activity](http://amigo.geneontology.org/cgi-bin/amigo/go.cgi?action=query&view=query&query=GO:0015075&search_constraint=terms) | GO:0015075 | 0.00146 |
| [Kinase activity](http://amigo.geneontology.org/cgi-bin/amigo/go.cgi?action=query&view=query&query=GO:0016301&search_constraint=terms) | GO:0016301 | 0.00288 |
| [Ras guanyl-nucleotide exchange factor activity](http://amigo.geneontology.org/cgi-bin/amigo/go.cgi?action=query&view=query&query=GO:0005088&search_constraint=terms) | GO:0005088 | 0.00414 |
| [Active transmembrane transporter activity](http://amigo.geneontology.org/cgi-bin/amigo/go.cgi?action=query&view=query&query=GO:0022804&search_constraint=terms) | GO:0022804 | 0.00490 |
| [Guanyl-nucleotide exchange factor activity](http://amigo.geneontology.org/cgi-bin/amigo/go.cgi?action=query&view=query&query=GO:0005085&search_constraint=terms) | GO:0005085 | 0.00604 |
| [Motor activity](http://amigo.geneontology.org/cgi-bin/amigo/go.cgi?action=query&view=query&query=GO:0003774&search_constraint=terms) | GO:0003774 | 0.00633 |
| [Protein kinase activity](http://amigo.geneontology.org/cgi-bin/amigo/go.cgi?action=query&view=query&query=GO:0004672&search_constraint=terms) | GO:0004672 | 0.01224 |
| [Transmembrane transporter activity](http://amigo.geneontology.org/cgi-bin/amigo/go.cgi?action=query&view=query&query=GO:0022857&search_constraint=terms) | GO:0022857 | 0.01403 |
| [Transporter activity](http://amigo.geneontology.org/cgi-bin/amigo/go.cgi?action=query&view=query&query=GO:0005215&search_constraint=terms) | GO:0005215 | 0.03076 |
| [Phosphotransferase activity, alcohol group as acceptor](http://amigo.geneontology.org/cgi-bin/amigo/go.cgi?action=query&view=query&query=GO:0016773&search_constraint=terms) | GO:0016773 | 0.03110 |
| [Substrate-specific transmembrane transporter activity](http://amigo.geneontology.org/cgi-bin/amigo/go.cgi?action=query&view=query&query=GO:0022891&search_constraint=terms) | GO:0022891 | 0.03628 |
| [Protein tyrosine kinase activity](http://amigo.geneontology.org/cgi-bin/amigo/go.cgi?action=query&view=query&query=GO:0004713&search_constraint=terms) | GO:0004713 | 0.03830 |
| [Ion binding](http://amigo.geneontology.org/cgi-bin/amigo/go.cgi?action=query&view=query&query=GO:0043167&search_constraint=terms) | GO:0043167 | 0.03847 |

Go: Gene ontology.

**Supplementary Table S2b Rich pathways of KEGG analysis of genes selected by screened SNPs.**

| **Exon with pathway annotation** | **Pathway ID** | **Corrected P-value** |
| --- | --- | --- |
| [Focal adhesion](file:///D:/%E6%9C%9D%E9%98%B3%E7%89%8C%E6%96%87%E7%AB%A0/%E5%85%A8%E5%9F%BA%E5%9B%A0%E7%BB%84%E5%A4%96%E6%98%BE%E5%AD%90%E6%B5%8B%E5%BA%8F/%E6%89%80%E6%9C%89SNP%E9%A2%91%E7%8E%87/control-vs-case/Pathway/Rich.html#gene1) | ko04510 | 1.707701e-05 |
| Foxo signaling pathway | Ko07201 | 4.839482e-05 |
| [Hypertrophic cardiomyopathy (HCM)](file:///D:/%E6%9C%9D%E9%98%B3%E7%89%8C%E6%96%87%E7%AB%A0/%E5%85%A8%E5%9F%BA%E5%9B%A0%E7%BB%84%E5%A4%96%E6%98%BE%E5%AD%90%E6%B5%8B%E5%BA%8F/%E6%89%80%E6%9C%89SNP%E9%A2%91%E7%8E%87/control-vs-case/Pathway/Rich.html#gene2) | ko05410 | 5.686038e-05 |
| [Amoebiasis](file:///D:/%E6%9C%9D%E9%98%B3%E7%89%8C%E6%96%87%E7%AB%A0/%E5%85%A8%E5%9F%BA%E5%9B%A0%E7%BB%84%E5%A4%96%E6%98%BE%E5%AD%90%E6%B5%8B%E5%BA%8F/%E6%89%80%E6%9C%89SNP%E9%A2%91%E7%8E%87/control-vs-case/Pathway/Rich.html#gene3) | ko05146 | 1.505625e-04 |
| [Dilated cardiomyopathy](file:///D:/%E6%9C%9D%E9%98%B3%E7%89%8C%E6%96%87%E7%AB%A0/%E5%85%A8%E5%9F%BA%E5%9B%A0%E7%BB%84%E5%A4%96%E6%98%BE%E5%AD%90%E6%B5%8B%E5%BA%8F/%E6%89%80%E6%9C%89SNP%E9%A2%91%E7%8E%87/control-vs-case/Pathway/Rich.html#gene4) | ko05414 | 2.206109e-04 |
| [Vascular smooth muscle contraction](file:///D:/%E6%9C%9D%E9%98%B3%E7%89%8C%E6%96%87%E7%AB%A0/%E5%85%A8%E5%9F%BA%E5%9B%A0%E7%BB%84%E5%A4%96%E6%98%BE%E5%AD%90%E6%B5%8B%E5%BA%8F/%E6%89%80%E6%9C%89SNP%E9%A2%91%E7%8E%87/control-vs-case/Pathway/Rich.html#gene5) | ko04270 | 3.751683e-03 |
| [Axon guidance](file:///D:/%E6%9C%9D%E9%98%B3%E7%89%8C%E6%96%87%E7%AB%A0/%E5%85%A8%E5%9F%BA%E5%9B%A0%E7%BB%84%E5%A4%96%E6%98%BE%E5%AD%90%E6%B5%8B%E5%BA%8F/%E6%89%80%E6%9C%89SNP%E9%A2%91%E7%8E%87/control-vs-case/Pathway/Rich.html#gene6) | ko04360 | 3.976980e-03 |
| [Tight junction](file:///D:/%E6%9C%9D%E9%98%B3%E7%89%8C%E6%96%87%E7%AB%A0/%E5%85%A8%E5%9F%BA%E5%9B%A0%E7%BB%84%E5%A4%96%E6%98%BE%E5%AD%90%E6%B5%8B%E5%BA%8F/%E6%89%80%E6%9C%89SNP%E9%A2%91%E7%8E%87/control-vs-case/Pathway/Rich.html#gene7) | ko04530 | 5.498337e-03 |
| [Regulation of actin cytoskeleton](file:///D:/%E6%9C%9D%E9%98%B3%E7%89%8C%E6%96%87%E7%AB%A0/%E5%85%A8%E5%9F%BA%E5%9B%A0%E7%BB%84%E5%A4%96%E6%98%BE%E5%AD%90%E6%B5%8B%E5%BA%8F/%E6%89%80%E6%9C%89SNP%E9%A2%91%E7%8E%87/control-vs-case/Pathway/Rich.html#gene8) | ko04810 | 5.498337e-03 |
| [ABC transporters](file:///D:/%E6%9C%9D%E9%98%B3%E7%89%8C%E6%96%87%E7%AB%A0/%E5%85%A8%E5%9F%BA%E5%9B%A0%E7%BB%84%E5%A4%96%E6%98%BE%E5%AD%90%E6%B5%8B%E5%BA%8F/%E6%89%80%E6%9C%89SNP%E9%A2%91%E7%8E%87/control-vs-case/Pathway/Rich.html#gene9) | ko02010 | 5.498337e-03 |
| [Arrhythmogenic right ventricular cardiomyopathy (ARVC)](file:///D:/%E6%9C%9D%E9%98%B3%E7%89%8C%E6%96%87%E7%AB%A0/%E5%85%A8%E5%9F%BA%E5%9B%A0%E7%BB%84%E5%A4%96%E6%98%BE%E5%AD%90%E6%B5%8B%E5%BA%8F/%E6%89%80%E6%9C%89SNP%E9%A2%91%E7%8E%87/control-vs-case/Pathway/Rich.html#gene10) | ko05412 | 1.087981e-02 |
| [ECM-receptor interaction](file:///D:/%E6%9C%9D%E9%98%B3%E7%89%8C%E6%96%87%E7%AB%A0/%E5%85%A8%E5%9F%BA%E5%9B%A0%E7%BB%84%E5%A4%96%E6%98%BE%E5%AD%90%E6%B5%8B%E5%BA%8F/%E6%89%80%E6%9C%89SNP%E9%A2%91%E7%8E%87/control-vs-case/Pathway/Rich.html#gene11) | ko04512 | 1.178052e-02 |
| [Pathways in cancer](file:///D:/%E6%9C%9D%E9%98%B3%E7%89%8C%E6%96%87%E7%AB%A0/%E5%85%A8%E5%9F%BA%E5%9B%A0%E7%BB%84%E5%A4%96%E6%98%BE%E5%AD%90%E6%B5%8B%E5%BA%8F/%E6%89%80%E6%9C%89SNP%E9%A2%91%E7%8E%87/control-vs-case/Pathway/Rich.html#gene12) | ko05200 | 1.244204e-02 |
| [Adherens junction](file:///D:/%E6%9C%9D%E9%98%B3%E7%89%8C%E6%96%87%E7%AB%A0/%E5%85%A8%E5%9F%BA%E5%9B%A0%E7%BB%84%E5%A4%96%E6%98%BE%E5%AD%90%E6%B5%8B%E5%BA%8F/%E6%89%80%E6%9C%89SNP%E9%A2%91%E7%8E%87/control-vs-case/Pathway/Rich.html#gene13) | ko04520 | 5.309787e-02 |
| [Dorso-ventral axis formation](file:///D:/%E6%9C%9D%E9%98%B3%E7%89%8C%E6%96%87%E7%AB%A0/%E5%85%A8%E5%9F%BA%E5%9B%A0%E7%BB%84%E5%A4%96%E6%98%BE%E5%AD%90%E6%B5%8B%E5%BA%8F/%E6%89%80%E6%9C%89SNP%E9%A2%91%E7%8E%87/control-vs-case/Pathway/Rich.html#gene14) | ko04320 | 5.309787e-02 |

KEGG: Kyoto Encyclopediaof Genes and Genomes.
