# Supplementary material for: Genetic variants in Forkhead box O1 associated with predisposition to sepsis in a Chinese Han population
Source: BMC Infect Dis. 2019 Sep 6;19:781. doi: 10.1186/s12879-019-4330-7 (PMC6731606; doi:10.1186/s12879-019-4330-7)
Supplement: Supplementary file 3 — Table S3. The information of SNPs selected for further validation. (DOCX 14 kb) [file 12879_2019_4330_MOESM3_ESM.docx]

**Supplementary Table S3. The information of SNPs selected for further validation**

| **Chromosome ID** | **Position** | **SNP ID** | **dbSNP**  **/novel** | **Ref** | **Gene name** | **Number** |
| --- | --- | --- | --- | --- | --- | --- |
| chr1 | 158225019 | rs2269715 | dbSNP; | ref=C; | CD1A | 7 |
| chr4 | 88904186 | rs1126772 | dbSNP; | ref=A; | SPP1 | 6 |
| chr7 | 94054194 | rs41317734 | dbSNP; | ref=C; | COL1A2 | 6 |
| chr7 | 94055885 | rs62464631 | dbSNP; | ref=G; | COL1A2 | 6 |
| chr14 | 95107145 | rs56952063 | dbSNP; | ref=T; | SERPINA13 | 6 |
| chr13 | 40565575 | rs2721068 | dbSNP; | ref=C | FOXO1 | 6 |
| chr13 | 40565740 | rs17446614 | dbSNP; | ref=A | FOXO1 | 6 |

Ref: reference allele of the SNP position, always uses alleles at positive strand of reference; Number: The number of sepsis patients who could be detected this SNP site.
