# Supplementary material for: Genetic variants in Forkhead box O1 associated with predisposition to sepsis in a Chinese Han population
Source: BMC Infect Dis. 2019 Sep 6;19:781. doi: 10.1186/s12879-019-4330-7 (PMC6731606; doi:10.1186/s12879-019-4330-7)
Supplement: Supplementary file 4 — Table S4. rs2269715 association with mortality of sepsis patients (n=156, adjusted by sex and age). (DOCX 14 kb) [file 12879_2019_4330_MOESM4_ESM.docx]

**Supplementary Table S4. rs2269715 association with mortality of sepsis patients (n=156, adjusted by sex and age).**

| Model | Genotype | Survivors | Non-survivors | OR(95%CI) | p value | AIC | BIC |
| --- | --- | --- | --- | --- | --- | --- | --- |
| Codominant | C/C | 38 (44.71%) | 20 (28.17%) | 1 | 0.22 | -234.98 | -222.78 |
|  | C/G | 35 (41.18%) | 44(61.98%) | 1.13(0.34, 3.7) |  |  |  |
|  | G/G | 12 (14.12%) | 7(9.86%) | 2.05(0.64, 6.56) |  |  |  |
| Dominant | C/C | 38 (44.71%) | 20 (28.17%) | 1 | 0.22 | -222.64 | -210.44 |
|  | C/G-G/G | 47(55.29%) | 51 (71.83%) | 0.64(0.31, 1.31) |  |  |  |
| Recessive | C/C-C/G | 73(85.88%) | 64(90.14%) | 1 | 0.44 | -235.36 | -223.16 |
|  | G/G | 12 (14.12%) | 7(9.86%) | 1.56(0.51, 4.74) |  |  |  |
| Overdominant | C/C-G/G | 50(58.83%) | 27 (38.02%) | 1 | 0.08 | -238.10 | -225.90 |
|  | C/G | 35 (41.18%) | 44(61.98%) | 0.53(0.26, 1.09) |  |  |  |

OR: Odd ratio; CI: Confidential interval; AIC: Akaike’s Information Criterion; BIC: Bayesian Information Criterion.
